# CRISPR/Cas9 engineering of a KIM-1 reporter human proximal tubule cell line

**DOI:** 10.1371/journal.pone.0204487

**Published:** 2018-09-27

**Authors:** Ruth Ann Veach, Matthew H. Wilson

**Affiliations:** 1 Department of Veterans Affairs, Nashville, Tennessee, United States of America; 2 Department of Medicine, Division and Nephrology and Hypertension, Vanderbilt University Medical Center, Nashville, Tennessee, United States of America; 3 Department of Pharmacology, Vanderbilt University, Nashville, Tennessee, United States of America; Tokushima University Graduate School, JAPAN

## Abstract

We used the CRISPR/Cas9 system to knock-in reporter transgenes at the kidney injury molecule-1 (KIM-1) locus and isolated human proximal tubule cell (HK-2) clones. PCR verified targeted knock-in of the luciferase and eGFP reporter at the KIM-1 locus. HK-2-KIM-1 reporter cells responded to various stimuli including hypoxia, cisplatin, and high glucose, indicative of upregulation of KIM-1 expression. We attempted using CRISPR/Cas9 to also engineer the KIM-1 reporter in telomerase-immortalized human RPTEC cells. However, these cells demonstrated an inability to undergo homologous recombination at the target locus. KIM-1-reporter human proximal tubular cells could be valuable tools in drug discovery for molecules inhibiting kidney injury. Additionally, our gene targeting strategy could be used in other cell lines to evaluate the biology of KIM-1 *in vitro* or *in vivo*.

## Introduction

Acute kidney injury is associated with high mortality; however, current therapy consists of mainly supportive care [1]. Kidney injury molecule-1 (KIM-1) was identified as a highly upregulated transcript in injured kidneys *in vivo* [2]. KIM-1 has been found to be a useful biomarker for kidney injury [3, 4]. *In vivo*, KIM-1 can be upregulated in proximal tubular cells in response to a variety of injuries [5, 6]. A greater understanding of what regulates the KIM-1 locus could lead to more insight into kidney injury. Additionally, drugs or interventions that block KIM-1 increase might be potentially therapeutic in the setting of kidney injury.

Previous investigators have created a bacterial artificial chromosome containing the KIM-1 promoter driving expression of a reporter transgene in mouse proximal tubular cells [6]. However, mice are different than humans, and kidney injury in mice and potential therapeutic interventions does not always translate to humans [7]. For these reasons, we sought to genome engineer human proximal tubule KIM-1-reporter cells.

The CRISPR/Cas9 system (clustered, regularly interspaced, short palindromic repeats (CRISPR)–CRISPR-associated protein 9) has enabled genome engineering in human cells. However, the use of CRISPR/Cas9 in the kidney has not yet been fully realized [8]. CRISPR/Cas9 enables user-selected genome engineering of target loci. A variety of approaches include target loci knockout, targeted insertion, gene upregulation, gene downregulation, and gene tagging [9]. We chose to use the CRISPR/Cas9 system to target insertion of reporter genes at the KIM-1 locus in human cells and evaluated these cells in response to known upregulators of KIM-1 *in vitro* and *in vivo* [10, 11].

## Materials and methods

### Targeting the KIM-1 locus

The gRNA (AGTCGTTGTCGTTGGAACAGTGG) was determined using CHOPCHOP (https://chopchop.rc.fas.harvard.edu/) and was cloned into the pX330-U6-Chimeric_BB-CBh-hSpCas9 vector [12]. Genomic DNA was isolated from HK-2 cells using a DNeasy kit (Qiagen, Valencia, CA) and 1 kb of upstream and downstream homologous DNA was amplified for cloning by PCR. Homologous DNA sequences were then subcloned into the PrecisionX HR targeting vector HR150PA-1 [GFP-T2A-Luc-pA-EF1α-RFP-T2A-Puro-MCS] (System Biosciences, Palo Alto, CA) using an In-Fusion kit (Clontech, Mountain View, CA). This vector is designed to direct in-frame fusion of GFP-T2A-Luciferase to an exon or the 3’ end of the chosen gene. Luciferase is expressed as a separate protein for assessment of gene expression. DNA sequencing was used to confirm the sequence of all DNA vectors. All primer sequences are listed in Table 1.

**Table 1:** Primer sequences.

Name	Sequence	use
KIM1upF	GACGGCCAGTGAATTCGGCCGTTGCTGTTTCTTAATGA	Infusion primer for homology domain
KIM1upR	CTTCATGGCGGGCATGAATTCGCTCGTTCGAACAGTCGTGACG	Infusion primer for homology domain
KIM1downF	AACCTAGATCGGATCGTTCCAATGACGACTGTTCCAACG	Infusion primer for homology domain
KIM1downR	CAGTCGACGGGGATCCTACCCAGGCTAGAGTGCAGTGGTG	Infusion primer for homology domain
KIM1geneupF	CGGTGGTGTGATTGCTACTCA	PCR validation of genome engineering
GFP_R	ctcgaactccacgccgttca	PCR validation of genome engineering
KIM1genednR	ACTCAGTGCTGATAATAGGAGGCA	PCR validation of genome engineering and Cre deletion
HR150PAseqdnF2	AGTCTATCAGAAGCTATCTGGTCTCC	PCR validation of genome engineering and Cre deletion
FlucF	TGTGTTTGTGGACGAAGTACCGAA	PCR validation of Cre deletion
Kim1dnHR_R	ACCTGGTTCATGGTTCTGCC	PCR validation of Cre deletion
hRPP30LWF	GGAGAGAGGATTGCTTGCGT	PCR validation of genomic DNA integrity
LW-RPR	GAGCGGCTGTCTCCACAAGT	PCR validation of genomic DNA integrity

### Cell culture and transfection

HK-2 cells [13] were cultured in DMEM/F12 medium supplemented with 5μg/mL insulin, 5μg/mL transferrin and 5ng/mL sodium selenite solution, 100U/mL penicillin and 100g/mL streptomycin solution, 0.1μM hydrocortisone (Sigma, St. Louis, MO), 2nmol/L L-glutamine and 10% fetal calf serum (HK-2CM) at 37°C with 5% CO_2_, and passaged at 2 x 10^5^/well to a 6 well plate for transfection. Cells were transfected with the pX330-U6-Chimeric_BB-CBh-hSpCas9 vector containing the gRNA and the GFP-T2A-Luc-pA-EF1α-RFP-T2A-Puro-MCS targeting vector with homology domains the following day using FuGENE 6 according to the manufacturer’s instructions (Promega, Madison, WI). RFP-positive clones were selected in HK-2 CM supplemented with 1–2 μg/mL puromycin. HK-2-KIM-1 reporter cells were infected with Adenovirus expressing Cre recombinase in the supernatant of HEK293-infected cells. Each 10 cm dish of cells at 50% confluency was infected with 1.65 x 10^12^ PFU on 3 consecutive days. For infection, cells were incubated with virus diluted in un-supplemented DMEM/F12 at 37°C with 5% CO_2_. After 5 hr, HK-2CM was added and cells were monitored for loss of RFP expression. RPTEC/TERT1 cells (kindly provided by Dr. William Fissell, Vanderbilt University Medical Center) [14] were cultured in serum-free DMEM/Ham’s F-12 (1:1) supplemented with 4 mM L-glutamine, 10 mM HEPES buffer, 5 nM triiodothyronine, 10 ng/ml recombinant human EGF, 3.5 μg/ml ascorbic acid, 5 μg/ml transferrin, 5 μg/ml insulin, 25 ng/ml prostaglandin E1, 25 ng/ml hydrocortisone, and 8.65 ng/ml sodium selenite (RPTEC-CM) at 37°C with 5% CO_2_, and passaged at 4 x 10^5^/well to a 6 well plate for transfection. RPTEC/TERT1 cells were transfected with FuGENE 6, jetPRIME (VWR, Radnor PA), or TransfeX (ATCC, Manassas, VA) the following day according to the manufacturer’s instructions. Clones were isolated in RPTEC-CM supplemented with 5–10% bovine growth serum (HyClone), 100μg/ml G418 and 3 μg/mL puromycin.

### Verification of genome engineering

Genomic DNA was isolated from puromycin-resistant clones using a DNeasy kit. PCR was performed using primers binding upstream or downstream of the genomic homology domains and within the gene tagging sequence to confirm insertion of the targeting cassette into the KIM-1 locus and Cre deletion of the RFP-T2A-Puro cassette (Table 1). PCR products were subjected to gel electrophoresis and visualized using ethidium bromide staining. Primers binding human ribonuclease P protein subunit p30 (a ubiquitous protein expressed in human cells) were used to confirm the integrity of genomic DNA when the absence of PCR products was the defining criteria for insertion or deletion. PCR products of the correct size were sequenced to confirm in-frame insertion of the gene tagging GFP-T2A-LUC sequence.

### Imaging of KIM-1 reporter cells

KIM-1 reporter cells were imaged on cell culture plates for RFP expression using a ZOE Fluorescent cell imager (Bio-rad, Hercules, CA). Reporter cells were imaged for luciferase expression using an IVIS Spectrum bioluminescent and fluorescent imaging system (PerkinElmer, Waltham, MA). Bioluminescent images were analyzed using Living Image Software (PerkinElmer).

### Evaluation KIM-1 reporter cell response to various stimuli

Verified HK-2-KIM-1 reporter cells were subjected to hypoxia (1% O_2_) using an InvivO_2_ hypoxia chamber (Baker, Sanford, ME). For measuring response to cisplatin, reporter clones cultured as described above in 12 well plates. When cells were 50–60% confluent, they were changed to medium without serum (SF-D/F) for 12 hrs. Cells were subsequently stimulated with cisplatin (0, 3 and 10 μM) in SF-D/F for 12 h. Cells were then changed to fresh SF-D/F and incubated for an additional 50–55 h (~ 65 h total). At the end of the experiment, luciferin was added (300 μg/mL) to triplicate wells each and images were captured on an IVIS as described above. Cells from one well each condition (not used for luciferase imaging) were harvested by trypsinization and counted (including cells in media) and all were determined to be >90% viable by trypan blue). For measuring response to glucose, clones were changed to DMEM as above but with low glucose (5.6 mM) for 12 hrs. Cells were subsequently serum-starved in DMEM with low glucose without serum for 12 hr, and then stimulated with 30.6 and 43.1 mM glucose 48 hr. Cells were imaged and viability was determined as described above.

### Statistical analysis

One way analysis of variance (ANOVA) followed by a Bonferroni post-test was used for multiple comparisons between groups studied.

## Results

### Genome engineering a reporter at the KIM-1 locus in human proximal tubular cells

**Fig 1:**
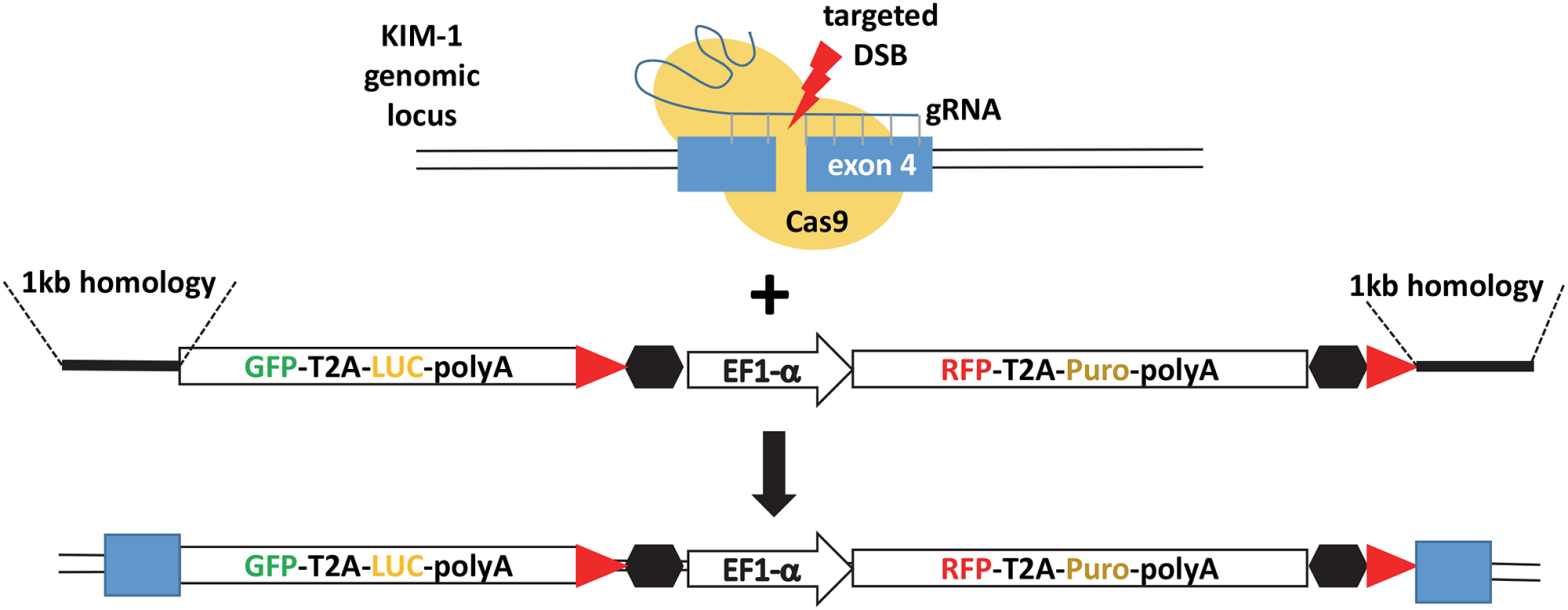
Schematic of Cas9-mediated homologous recombination targeting vector-mediated genome engineering of human proximal tubule cells at the KIM-1 locus. Cas9-gRNA mediated DNA break (red lightning bolt) facilitates integration via homology arms. GFP-T2A-Luc is fused in-frame into exon 4. Floxed (red triangles) RFP-T2A-Puro permits selection of targeted cells and the selection cassette can be removed with Cre recombinase, if desired. The RFP-T2A-Puro cassette is flanked by insulator elements (black hexagons). GFP, green fluorescent protein; T2A, self-cleaving peptide sequence; LUC, firefly luciferase; polyA, polyadenylation sequence; EF1a, EF1a constitutive promoter; RFP, red fluorescent protein; Puro, puromycin N-acetyl-transferase.

The human KIM-1 locus (also named the hepatitis A virus cellular receptor 1 (HAVCR1)) is located on chromosome 5 containing multiple possible transcripts. In order to ensure proper genomic tagging of the KIM-1 locus, we chose a gRNA targeting an exon of all possible isoforms (exon 4). We chose a targeting vector that would enable fusion of GFP-T2A-luciferase into exon 4 permitting transcription and translation of in-frame GFP fusion and cellular production of luciferase to allow bioluminescence as a readout of KIM-1 expression (Fig 1). Our vector also contained an independent transgene cassette for RFP expression and puromycin resistance, allowing confirmation of transfection efficiency and isolation of cells which had undergone genome engineering at the target locus. The RFP-T2A-Puro cassette was flanked by insulator elements so that the EF1α promoter would not affect expression from the KIM-locus.

**Fig 2:**
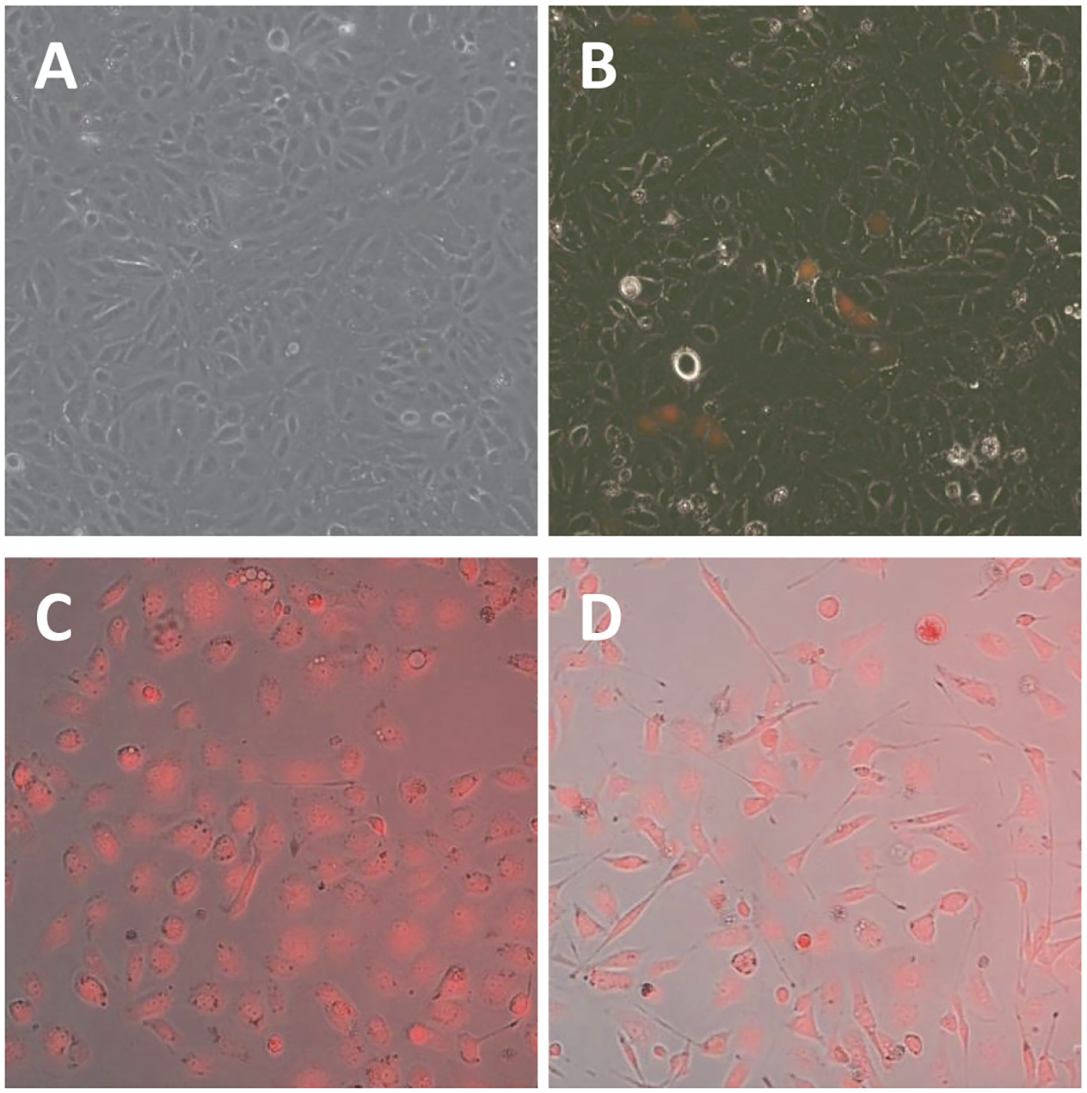
Stable transfection and isolation of HK-2 KIM1-reporter clones. Unmodified cells (**A**) were transfected with the gene targeting vector and active Cas9 and evaluated for RFP expression on day 2 (**B**). Puromycin selection permitted isolation of HK-2 clones with 100% of cells expressing RFP (**C** and **D**).

**Fig 3:**
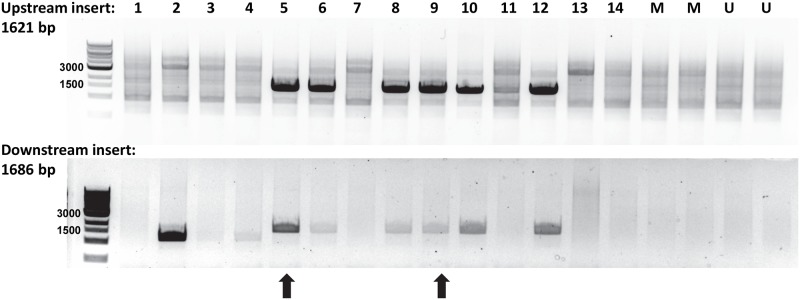
Verification of gene tagging in generating KIM-1-reporter cell lines. PCR was used to verify proper gene tagging by amplifying the genomic sequence upstream and downstream of the homology arms in the targeting vector with additional primers within the integrated DNA sequence. Correct upstream PCR results in a band of 1621 bp whereas correct downstream amplification results in a band of 1686 bp. 16 clones were evaluated as well as mock transfected (M) and untransfected (U) cells. Clones 5 and 9 (black arrows) were chosen for further studies due to sequence verified in-frame gene tagging.

HK-2 cells are an immortalized human proximal tubule cell line. We transfected HK-2 cells with our targeting vector and active Cas9 and confirmed RFP expression (Fig 2A and 2B). We used puromycin selection to isolate clones with 100% RFP expression (Fig 2C and 2D). Clones could either have integrated our cassette at a non-target locus, or RFP expressing clones could have correctly inserted the gene targeting cassette into the KIM-1 locus. In order to verify proper gene targeting, we used PCR to amplify the genomic DNA segment both upstream and downstream of our targeting cassette (primers, Table 1). A positive PCR result would result only if primers amplified from genomic DNA at the target locus upstream of our homology targeting domain and amplified from within the non-homology transgene cassette. There were 32 clones screened by PCR for correct insertion at both the upstream and downstream genomic loci. Of these, 16 were positive for both insertions by PCR. Six of these PCR-positive clones were randomly chosen for sequencing of the upstream PCR product, but the correct sequence in frame could be confirmed in only two (Fig 3). These clones, 5 and 9, were selected for evaluation of KIM-1 response.

### KIM-1 reporter cells respond to stimuli

Although KIM-1 is expressed in response to a wide variety of injurious insults *in vivo*, cultured human proximal tubular cells have not always recapitulated *in vivo* response. This could be due to a variety of reasons, including experimental conditions. However, others have reported KIM-1 expression to be increased in response to hypoxia, cisplatin, and glucose in cultured proximal tubular cells [6, 10, 15].

**Fig 4:**
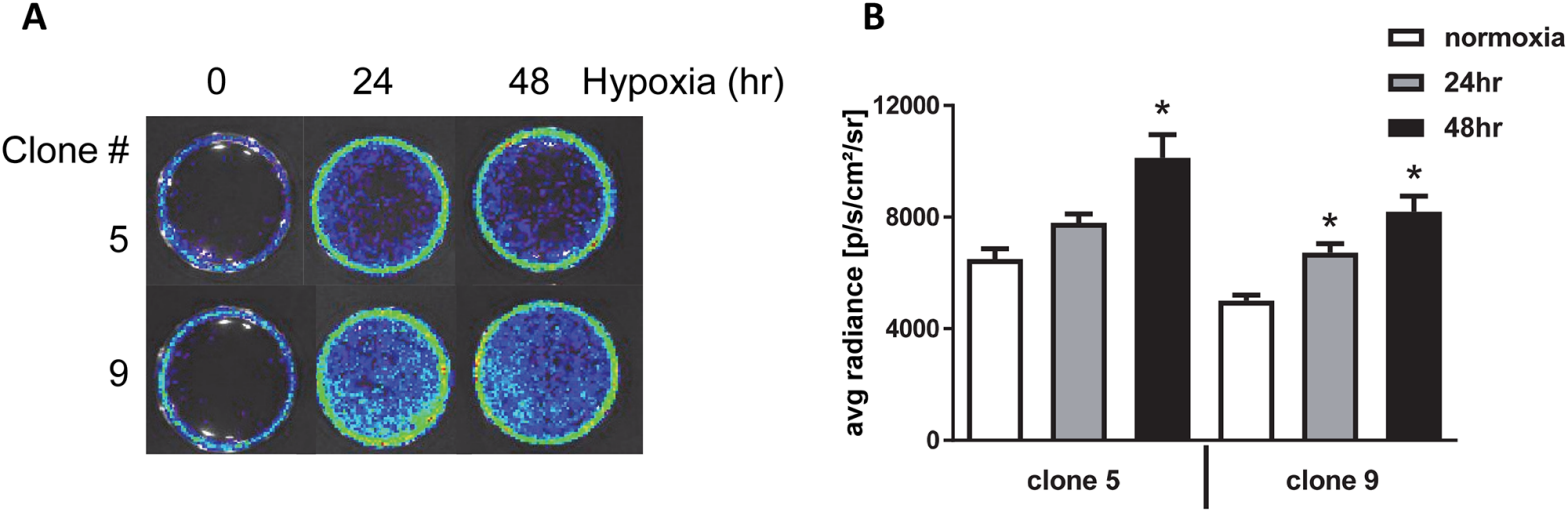
KIM-1 reporter cell line response to hypoxia. (**A**), clones 5 and 9 demonstrate increased luciferase expression via IVIS imaging in response to hypoxia (1% O_2_) over a course of 24–48 hours. (**B**), This hypoxic response was reproducible with little variation between clones. Cell viability at the time of imaging was >90%. *, p<0.05 via ANOVA followed by Bonferroni post-test (N = 3±SEM).

**Fig 5:**
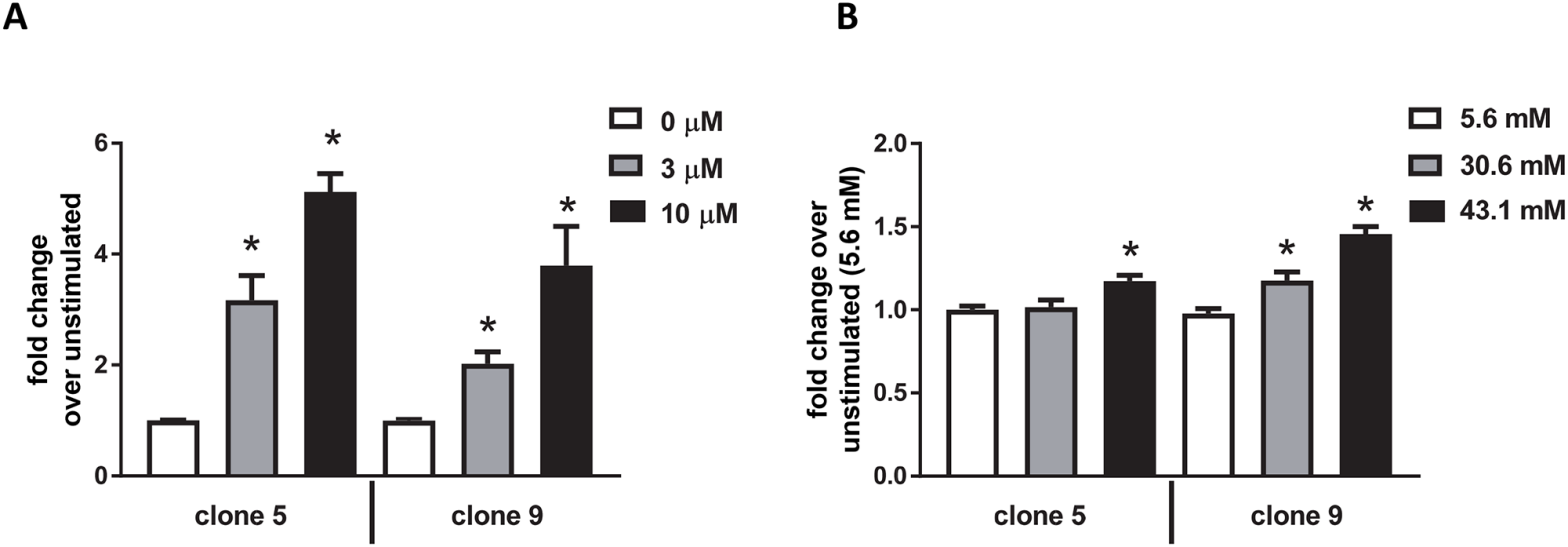
KIM-1 reporter cell line response to cisplatin and glucose. Clones 5 and 9 demonstrate increased luciferase expression via IVIS imaging in response to increasing concentrations of cisplatin (**A**) or glucose (**B**). Cell viability at the time of imaging was >90%. *, p<0.05 via ANOVA followed by Bonferroni post-test (N = 3±SEM).

Our reporter gene tagging vector (Fig 1) enables luciferase expression in response to increased expression of endogenous KIM-1. Therefore, we used bioluminescent imaging to evaluate and quantitate KIM-1 response to hypoxia using our genome-engineered cells that express luciferase in response to upregulation of the KIM-1 locus. We evaluated both clone 5 and clone 9 and observed increased luciferase expression after exposing KIM-1-reporter cells to hypoxic conditions (1% O_2_) for 24 and 48 hours (Fig 4A). On average, we observed a 27% and 59% increase in luciferase expression compared to cells cultured in normoxic conditions at 24 and 48hr of hypoxia, respectively, between both clones evaluated (Fig 4B). These results are consistent with what others have reported when evaluating KIM-1 using RT-PCR of KIM-1 RNA in hypoxic HK-2 cells [10]. We additionally evaluated our reporter cells in response to cisplatin and high glucose using previously published protocols [6, 15]. Both clones exhibited a 2–3 fold and 4–5 fold increase in luciferase expression when treated with 3μM or 10μM cisplatin respectively (Fig 5A). Response to glucose was less robust; however, we were able to observe a reproducible 20–40% increase in luciferase expression with higher glucose concentrations (Fig 5B). Our HK-2-KIM-1 reporter cells, therefore, represent a human proximal tubule KIM-1-reporter cell line.

**Fig 6:**
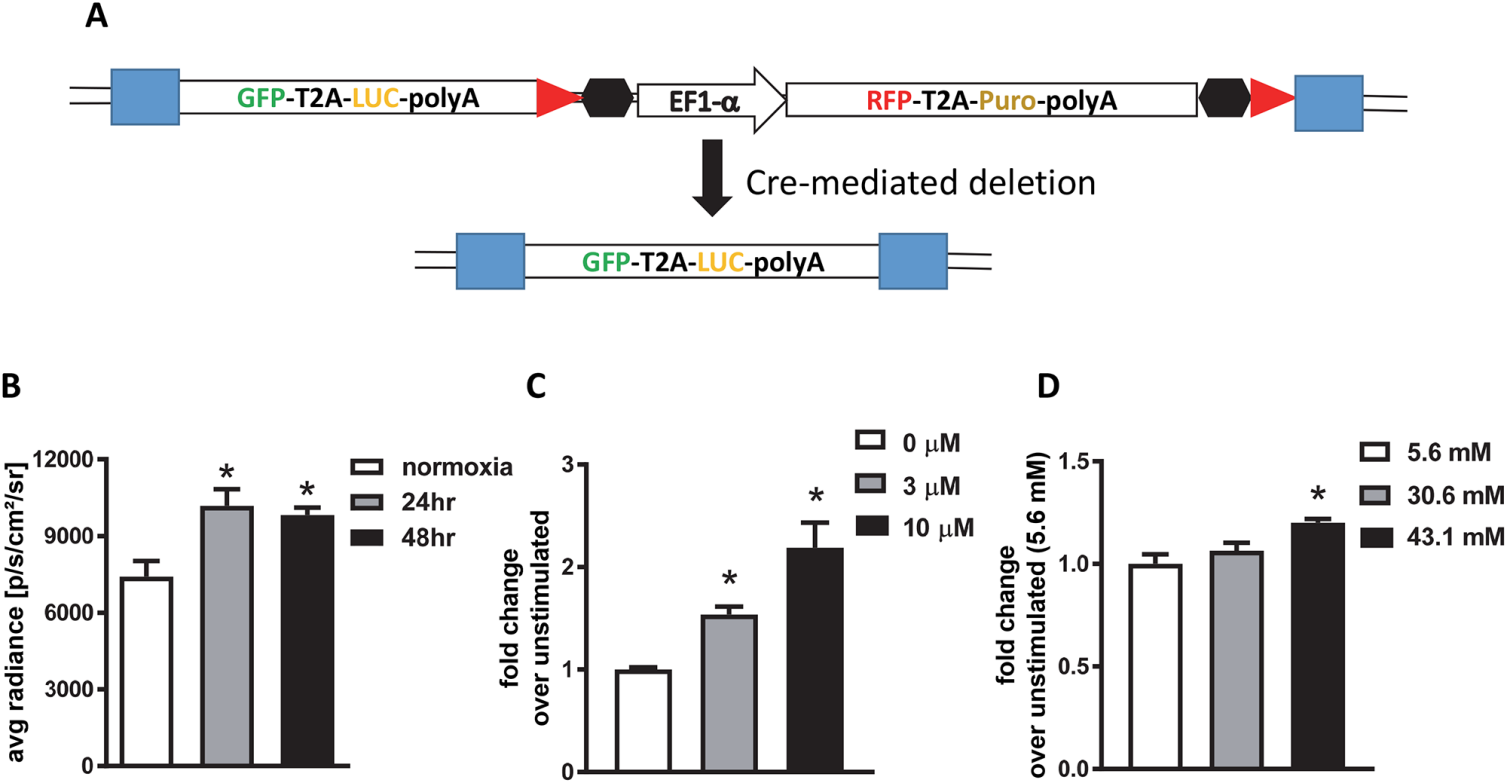
Clone 5 retains the response to stimuli after deletion of the RFP-T2A-Puro cassette with Cre recombinase. (**A**), schematic of cre-mediated deletion of RFP-T2A-Puro cassette at the genomic level. (**B–D**), RFP-T2A-Puro deleted cells response to hypoxia, cisplatin, and glucose respectively. Cell viability at the time of imaging was >90%. *, p<0.05 via ANOVA followed by Bonferroni post-test (N = 3±SEM).

We next tested whether deletion of the RFP-T2A-Puro selection cassette would have an impact on reporter activity (Fig 6A). Reporter cells were infected with adenovirus expressing Cre recombinase on 3 consecutive days. One week after infection, RFP expression was no longer detectable by fluorescence microscopy. Genomic DNA was isolated from infected cells to confirm deletion of the RFP-T2A-Puro selection cassette by PCR using primers binding in the luciferase-expressing region or between the LoxP sites and the downstream KIM-1 homology region or the downstream KIM-1 gene (Table 1). The sizes, or absence, of resulting PCR products were consistent with truncation due to Cre deletion. Cre-deleted clone 5 cellswere evaluated for luciferase expression in response to hypoxia, cisplatin, and glucose as before. These cells retained the response to all stimuli as demonstrated before deletion of the selection cassette (Fig 6B and 6C).

### Not all human proximal tubular cell lines permit genome engineering

**Fig 7:**
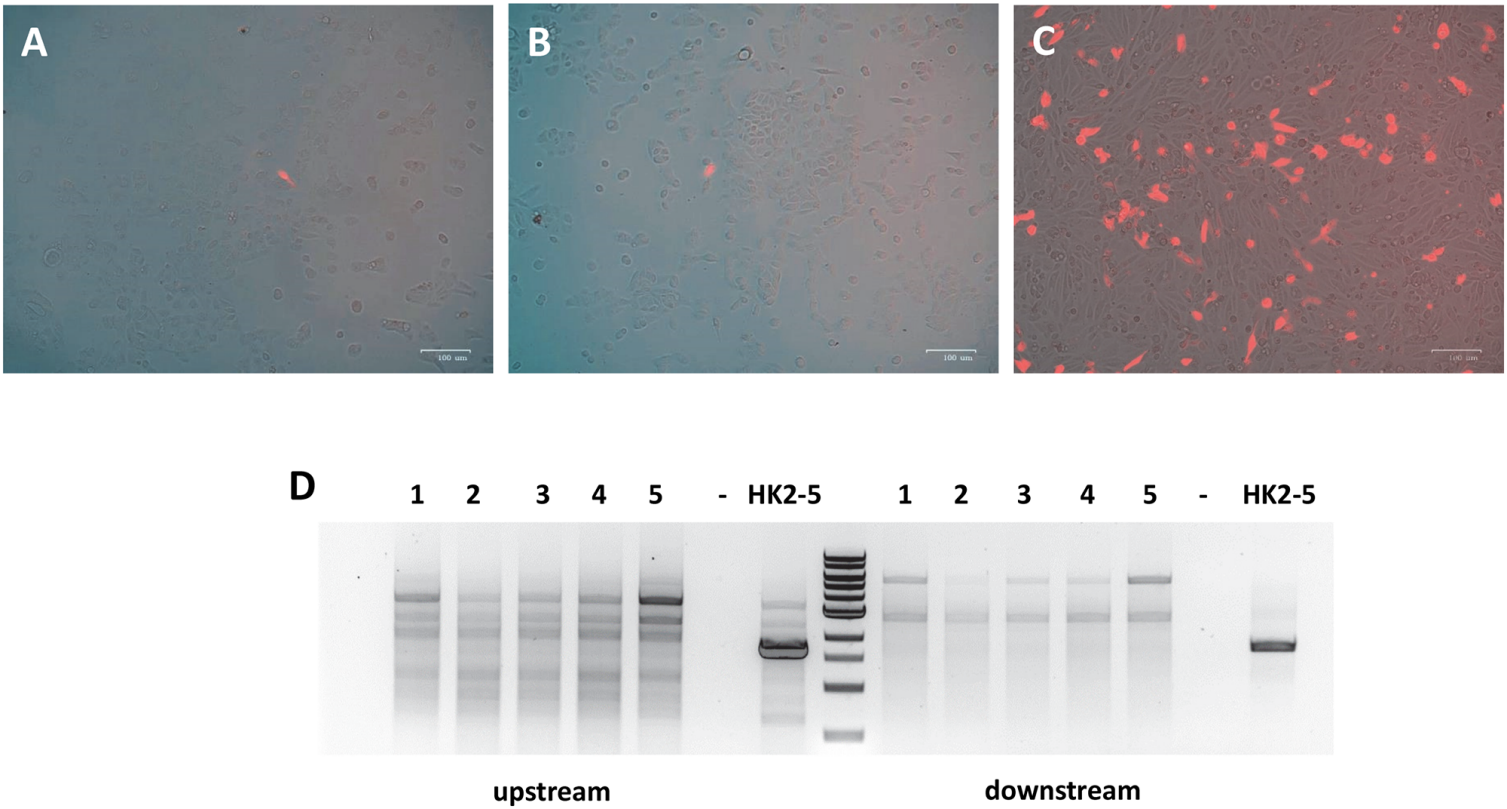
Lack of Cas9-mediated gene tagging of KIM-1 in RPTEC cells. RPTEC cells were transfected with jetPRIME (**A**), FuGENE-6 (**B**), and TransfeX (**C**) with TransfeX proving to be the most efficient. Despite good transfection efficiency, only 4 puromycin resistant clones were isolated. None of these clones demonstrated verified gene tagging via PCR (**D**). HK-2 clone 5 (HK-2-5) was used as a positive control. (-), untransfected cells.

RPTEC/TERT1 cells are another human proximal tubule cell line, immortalized with human telomerase. We attempted to use genome engineering to label the KIM-1 locus in these cells as well. We compared three different transfection reagents to determine the most efficient reagent for transfecting RPTEC/TERT1 cells. A comparison of jetPRIME, FuGENE 6, and TransfeX revealed TransfeX to be the most efficient (Fig 7A–7C). We subsequently used TransfeX to transfect our gene targeting vector (Fig 1) and active Cas9 into RPTEC/TERT1 cells. Despite good transfection efficiency (Fig 7C), it was difficult to isolate puromycin resistant clones. We evaluated the 4 clones we could isolate by PCR to validate for possible gene targeting efficiency as we had done in HK-2 cells. None of the 4 clones exhibited correct insertion of our targeting vector at the KIM-1 locus, indicating that RFP expression in these clones was due to random integration of the targeting vector and not targeted gene tagging (Fig 7D). Therefore, RPTEC/TERT1 cells appear to be not as amenable as HK-2 cells to gene tagging using homology dependent repair strategies.

## Discussion

We used CRISRP/Cas9 to genome engineer KIM-1-reporter human proximal tubular cell lines responsive to various stimuli. These KIM-1 reporter cells could subsequently be used to further elucidate the response of KIM-1 to hypoxia, cisplatin, or glucose, and perhaps even be used in drug discovery for interventions which block KIM-1 upregulation.

qRT-PCR KIM-1 assays have been used in a variety of cultured proximal tubular cell types to evaluate mRNA levels. qRT-PCR measures the steady state level of the transcript at the time point analyzed. This tells you very little about the overall transcriptional control of the gene. A genome engineered reporter cell line, however, reveals promoter activation or transcriptional regulation as the expression of the reporter transgene is dependent upon the endogenous promoter at the site of knock-in. We, therefore, chose to develop this alternative approach using genome engineering technology to look at the response of the KIM-1 genomic locus to various stimuli. Our readout may be different from using qRT-PCR because our readout is of luciferase protein rather than KIM-1 RNA, which can be affected by multiple variables including mRNA generation and breakdown rates.

Why was there a difference in gene tagging efficiency between HK-2 and RPTEC/TERT1 cells? The same targeting vectors were utilized between cell types and transfection efficiency appeared adequate. Non-homologous end joining (NHEJ) is the major double stranded break (DSB) repair pathway in proliferating and post-mitotic cells [16]. NHEJ is generally more efficient than homology directed repair (HDR) in mammalian species [17]. In order for CRISRP/Cas9 to be effective in gene tagging and creating reporter cell lines, the technology must create a DSB but HDR must also be working for user-selected gene tagging. Given that all cells do not undergo HDR at the same efficiency, the ability to gene tag a given locus between different cell lines or types may vary. This could be due to epigenetic factors or other reasons currently not well understood. Indeed, the ability of CRISPR/Cas9 to genome engineer cells varies between cell types even when using the same gRNA [18]. Interestingly, when using Cas9 to genome engineer human proximal tubular cells, we found HK-2 cells more amenable to homology directed integration of genetic cargo than RPTEC/TERT1 cells. Our results reveal that CRISPR/Cas9 technology can permit genome editing in HK-2 cells, which are widely used in kidney research.

Our genome engineering strategy could easily be adapted to other cell types, including human induced pluripotent stem cells (iPSCs). iPSCs have been differentiated into kidney organoids that do appear to upregulate KIM-1 in response to cisplatin [19]. Therefore, our gene tagging strategy, which has now been verified in cultured human proximal tubule cells, could be used to modify the KIM-1 locus in iPSCs. Engineered KIM-1 reporter iPSCs could then be differentiated into organoids, or even implanted into immunodeficient mice, enabling bioluminescent imaging of KIM-1 expression *in vitro* and *in vivo*. This could allow researchers to better evaluate both nephrotoxins and therapeutic interventions for preventing or alleviating nephrotoxicity. Given the current lack of therapeutic interventions for kidney injury, high throughput strategies aimed at evaluating KIM-1 expression response using KIM-1 reporter cells could lead to development of new therapies for kidney disease.
